# Diagnostic Concordance between Clinical Dementia Rating, Hindi Mental State Examination and Clinical Evaluation

**DOI:** 10.1002/alz70857_107816

**Published:** 2025-12-26

**Authors:** Jyothi M S Gowda, Aparna N P, Kavya K Kumar, Apurva Mittal, Himanshu Joshi, Subhashini K Rangarajan, Mathew Varghese, Sivakumar PT, Ganesan Venkatasubramanian, Paul M. Thompson, John P John

**Affiliations:** ^1^ National Institute of Mental Health and Neurosciences (NIMHANS), Bengaluru, Karnataka, India; ^2^ Multimodal Brain Image Analysis Laboratory (MBIAL), National Institute of Mental Health and Neurosciences (NIMHANS), Bengaluru, Karnataka, India; ^3^ St John's Medical College, Bengaluru, Karnataka, India; ^4^ Departmet of Psychiatry, National Institute of Mental Health and Neurosciences (NIMHANS), Bengaluru, Karnataka, India; ^5^ University of Southern California, Los Angeles, CA, USA; ^6^ Imaging Genetics Center, Mark and Mary Stevens Neuroimaging & Informatics Institute, University of Southern California, Marina del Rey, CA, USA; ^7^ Imaging Genetics Center, Mark and Mary Stevens Neuroimaging and Informatics Institute, Keck School of Medicine, University of Southern California, Marina del Rey, CA, USA; ^8^ Keck School of Medicine, University of Southern California, Los Angeles, CA, USA; ^9^ Multimodal Brain Image Analysis Laboratory, National Institute of Mental Health and Neurosciences (NIMHANS), Bengaluru, Karnataka, India; ^10^ National Institute of Mental Health And Neurosciences, Bengaluru, Karnataka, India

## Abstract

**Background:**

Clinical Dementia Rating (CDR) and Hindi Mental State Examination (HMSE) are ubiquitously used by clinicians and researchers in India for quantifying and classifying severity of cognitive impairment. he clinical dementia rating (CDR) scale measures cognitive impairment in 6 domains, including memory, orientation, judgment and problem solving, community affairs, home and hobbies, and personal care. It rates severity from 0 = normal to 3 = severe on the basis of functional and cognitive decline. The Hindi Mental Status Examination (HMSE), a 31‐point cognitive test designed for the Indian population, evaluates orientation, memory, attention, language, and visuospatial abilities. In this study, we examined the diagnostic concordance between CDR, HMSE and clinical evaluation (CE) by expert Geriatric Psychiatrists, in the NIH/NIA‐funded R01 project titled “India ENIGMA Initiative for Global Ageing and Mental Health” (R01AG060610).

**Method:**

Data from 85 participants enrolled in the project were used for this study. The participants were clinically evaluated and diagnosed by expert psychiatrists; while CDR and HMSE were administered by trained and certified research fellows. The classification based on CE [ HE (*n* = 47), MCI (*n* = 24), and AD (*n* = 14)] by expert psychiatrists was considered as the ‘gold standard’. The overall agreement among CDR, HMSE, and CE was assessed using Fleiss' multi‐rater kappa, while Cohen's weighted kappa was used to evaluate agreement within individual categories

**Result:**

Clinical ratings of 85 participants using CA, CDR and HMSE showed “moderate” agreement (Fleiss kappa = 0.556; Z=11.885; *p* <0.001) with each other. The agreement between CA and CDR was “very good” weighted kappa=0.954; Z=10.994; *p* <0.001), and agreement with HMSE was “moderate” (weighted kappa=0.483; Z=6.041; *p* <0.001). The agreement between HMSE and CDR was “moderate” (weighted kappa=0.482; Z=5.99, *p* <0.001).

Between all the three methods, the agreement on Healthy Elders was “very good” (weighted kappa=0.848; Z=9.311; *p* <0.001), agreement within the MCI category was “moderate” (weighted kappa=0.518; Z=6.171; *p* <0.001) and agreement within AD category was “good” (weighted kappa=0.784; Z=12.088; *p* <0.001)

**Conclusion:**

The results of the study highlight the excellent concordance between CDR‐derived diagnoses with CE‐based diagnoses, the gold standard, in this project that aims to examine the various factors contributing to the ‘brain age gap’ in AD and MCI in India.

